# HIITing Anxiety and Depression in Parkinson’s Disease and Multiple Sclerosis—A Study Protocol of a Transdiagnostic Randomized Controlled Trial (HersenFIT)

**DOI:** 10.3390/brainsci15090945

**Published:** 2025-08-29

**Authors:** Arianne S. Gravesteijn, Marc B. Rietberg, Vincent de Groot, Mark A. Hirsch, Tim Vanbellingen, Richard T. Jaspers, Chris Vriend, Wilma D. J. van de Berg, Odile A. van den Heuvel, Erwin E. H. van Wegen, on behalf of the HersenFIT Consortium

**Affiliations:** 1 Amsterdam UMC Location Vrije Universiteit Amsterdam, Rehabilitation Medicine, De Boelelaan 1117, 1081 HV Amsterdam, The Netherlands; a.gravesteijn@amsterdamumc.nl (A.S.G.); m.rietberg@amsterdamumc.nl (M.B.R.); v.degroot@amsterdamumc.nl (V.d.G.); 2 Amsterdam Neuroscience, Neuroinfection & Inflammation, 1081 HV Amsterdam, The Netherlands; 3 Amsterdam Movement Sciences, Rehabilitation & Development, 1081 HZ Amsterdam, The Netherlands; r.t.jaspers@vu.nl; 4 MS Center Amsterdam, 1081 HV Amsterdam, The Netherlands; 5 Carolinas Rehabilitation, Department of Physical Medicine and Rehabilitation, Charlotte, NC 28203, USA; 6 Wake Forest School of Medicine, Department of Orthopedic Surgery and Rehabilitation, Winston-Salem, NC 27157, USA; 7 Gerontechnology and Rehabilitation Group, University of Bern, 3012 Bern, Switzerland; tim.vanbellingen@unibe.ch; 8 VAMED Management & Services Schweiz AG, Research and Innovation, 8001 Zurich, Switzerland; 9 Laboratory of Myology, Department of Human Movement Science, Faculty of Behavioral and Movement Sciences, Location Vrije Universiteit Amsterdam, 1081 HV Amsterdam, The Netherlands; 10 Amsterdam Movement Sciences, Tissue Function & Regeneration, 1081 HZ Amsterdam, The Netherlands; 11 Amsterdam UMC Location Vrije Universiteit Amsterdam, Anatomy & Neurosciences, De Boelelaan 1117, 1081 HV Amsterdam, The Netherlands; c.vriend@amsterdamumc.nl (C.V.); oa.vandenheuvel@amsterdamumc.nl (O.A.v.d.H.); 12 Amsterdam UMC Location Vrije Universteit Amsterdam, Psychiatry, De Boelelaan 1117, 1105 AZ Amsterdam, The Netherlands; wdj.vandeberg@amsterdamumc.nl; 13 Amsterdam Neuroscience, Compulsivity Impulsivity & Attention, 1081 HV Amsterdam, The Netherlands; 14 Amsterdam Neuroscience, Neurodegeneration, 1081 HV Amsterdam, The Netherlands; 15 Amsterdam Neuroscience, Compulsivity, Impulsivity and Attention, 1081 HV Amsterdam, The Netherlands

**Keywords:** Parkinson’s disease, multiple sclerosis, high-intensity interval training, exercise, anxiety, depression

## Abstract

**Background/Objectives:** Parkinson’s disease (PD) and multiple sclerosis (MS) are neurological conditions that result in debilitating non-motor symptoms, such as anxiety and depression, which significantly reduce quality of life and often persist despite pharmacological treatment. As a result, effective alternative treatment strategies are needed. Exercise therapy—particularly aerobic training—has shown promise in alleviating non-motor symptoms, potentially through neuroplastic adaptations. However, traditional aerobic exercise is often time-consuming and monotonous, limiting long-term adherence. High-intensity interval training (HIIT) offers a time-efficient and potentially more engaging alternative, though its effects on non-motor symptoms in PD and MS remain understudied. **Methods**: This transdiagnostic randomized controlled trial will enroll 48 participants (24 PD, 24 MS) with clinically significant affective symptoms (hospital anxiety and depression scale [HADS] ≥ 8). The participants will be randomly assigned to one of three 8-week interventions: (1) HIIT, 5–6 intervals of 45 s of high-intensity cycling; (2) continuous aerobic training (CAT), 50 min of low-intensity cycling; (3) movement advice (MA), step goals, and physical education. The primary (affective symptoms) and secondary outcomes (cognition, fatigue, sleep, motor function) will be assessed at four time points: 4 and 1 weeks pre intervention, and 1 and 4 weeks post intervention. Weekly blood samples and pre/post brain imaging will be collected to study biofluid and MRI measures for potential neuroplasticity. Linear mixed models will analyze the time and group effects. **Discussion:** This trial will assess whether HIIT can more effectively improve non-motor and motor symptoms in PD and MS than CAT or MA. A multimodal approach will explore both the clinical outcomes and underlying mechanisms, informing scalable and engaging rehabilitation strategies.

## 1. Introduction

Parkinson’s disease (PD) and multiple sclerosis (MS) are chronic, complex, and highly disabling neurodegenerative disorders affecting the central nervous system. PD is primarily characterized by the progressive loss of dopaminergic neurons in the substantia nigra and widespread Lewy body pathology. Increasing evidence also implicates neuroinflammation in the pathogenesis and progression of PD [1]. In parallel MS is driven by autoimmune-mediated neuroinflammation and neurodegeneration, including axonal and neuronal loss [2,3,4]. Despite these distinct pathophysiological mechanisms and clinical courses, individuals with PD and MS experience overlapping characteristics.

While motor impairments such as gait disturbances and balance difficulties are commonly recognized in PD and MS [5,6], non-motor symptoms—including anxiety, depression, cognitive deficits, fatigue, and sleep disturbances—can be equally debilitating [7,8,9,10]. Approximately 30–50% of individuals with PD or MS experience significant affective symptoms during the disease course [11,12,13], which are associated with accelerated cognitive and physical decline, reduced quality of life, and impaired overall well-being [7,14]. Although often overshadowed by motor symptoms, the profound impact of non-motor symptoms on everyday life warrants targeted interventions.

The pathophysiological pathways in both PD and MS can contribute to anxiety and depression. These neurodegenerative diseases share common mechanisms that can underlie the development of affective symptoms, including neuroinflammation, HPA axis dysregulation, neurotransmitter dysfunction, and reduced neuroplasticity, which are also related to anxiety and depression [15]. In addition, anxiety and depression in PD and MS can be exacerbated by other disease-related symptoms such as sleep disturbances, which are common, and may overlap with affective symptoms [16]. Considering these similarities, a transdiagnostic approach—focusing on the shared mechanisms and symptoms across disorders rather than disease-specific features—may offer a more holistic and efficient strategy for managing anxiety and depression in PD and MS [17,18].

While pharmacological strategies remain a cornerstone in the management of PD and MS, they often fall short in fully addressing the wide range of motor and non-motor symptoms [4,19]. Furthermore, side effects and complex drug interactions may exacerbate symptom burden [20,21,22]. As a result, there is a growing interest in alternative approaches, with exercise therapy emerging as a particularly promising intervention [23,24,25,26].

Exercise is already well established in improving motor symptoms and a growing body of evidence highlights its benefits for affective disturbances such as anxiety and depression [24,27]. Among various exercise modalities, aerobic and strength training are the most extensively studied, with aerobic training showing particular promise in alleviating affective symptoms and promoting neuroprotective mechanisms [28,29]. Traditional continuous aerobic training (CAT)—characterized by prolonged low-to-moderate-intensity exercise—has demonstrated well established and robust effectiveness on physical fitness and motor and non-motor functions but may be time-consuming and monotonous, potentially limiting adherence. Alternatively, high-intensity interval training (HIIT), involving short bursts of vigorous activity alternated with recovery periods, has emerged as a time-efficient and engaging alternative which may be superior to CAT [30,31,32,33]. In both PD and MS, HIIT has been shown to be a safe and feasible intervention, with benefits for motor symptoms, fatigue, and physical fitness [34,35,36]. Beyond these benefits, HIIT may positively influence mood [37,38]. However, the current evidence, particularly regarding its effects on non-motor symptoms such as anxiety and depression, remains limited and inconclusive [39].

Furthermore, exercise intensity appears to be an important driver of neuroplastic adaptations; however the optimal intensity remains unclear [40]. Emerging research suggests that exercise may contribute to slowing disease progression through neuroprotective mechanisms [28,29]. These include the preservation of regional brain volumes—particularly in the hippocampus and cortical areas [41,42]—the enhancement of white matter integrity (e.g., increased fractional anisotropy) [43], and the modulation of resting state functional connectivity [44]. One frequently studied mechanism is the upregulation of neurotrophic factors, such as brain-derived neurotrophic factor (BDNF), which supports synaptic plasticity, learning, and memory [45,46]. Notably, reduced BDNF concentrations have been associated with affective symptoms, including anxiety and depression [47]. Exercise, and especially high-intensity exercise, has been shown to increase BDNF expression, even in populations with neurodegenerative conditions, and this upregulation may mediate improvements in mood as well as broader neuroplastic changes [48,49,50]. This growing evidence base provides a strong rationale for evaluating exercise as a therapeutic strategy targeting both non-motor symptoms and neurobiological outcomes.

Despite the growing interest, comparative studies evaluating the effects of HIIT and CAT on non-motor symptoms and neuroplasticity in PD and MS remain scarce [51,52]. Moreover, people with these conditions may face barriers to engage in structured HIIT and CAT exercise interventions, such as limited accessibility, fatigue, or lack of time, which highlights the need for more flexible alternatives [53]. Movement advice (MA), which involves personalized guidance to increase daily physical activity, offers a pragmatic, home-based approach that may improve feasibility and adherence. We are optimistic that evaluating MA alongside CAT and HIIT will provide valuable insights into the feasibility, effectiveness, and individualization of exercise-based interventions for managing non-motor symptoms in PD and MS. This randomized controlled trial aims to investigate the effects of HIIT on non-motor symptoms, particularly anxiety and depression, compared with CAT and MA in individuals with PD and MS. Secondary objectives include evaluating the impact of exercise on blood and MRI measures of neuroplasticity, neurodegeneration, and overall quality of life.

## 2. Materials and Methods

### 2.1. Study Design and Setting

**Figure 1:**
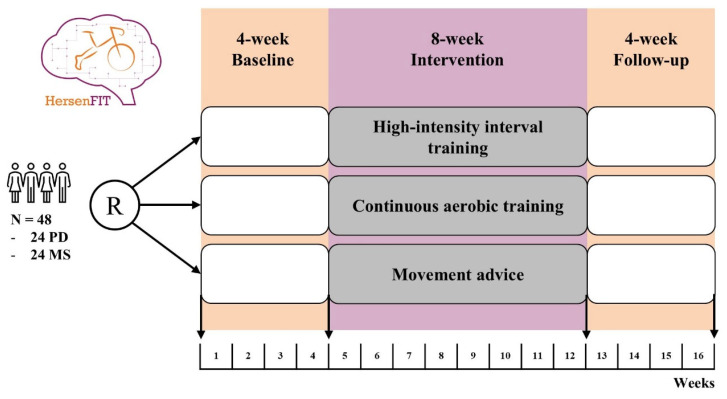
Study design. Abbreviations: PD, Parkinson’s disease; MS, multiple sclerosis.

This is a single-blind, monocenter, randomized trial, with serial baseline–intervention–follow-up periods and three parallel intervention arms (see Figure 1). The total study duration per participant is 16 weeks and it will be conducted at Amsterdam UMC. All participants will perform two baseline assessments four weeks apart (i.e., baseline period), followed by an eight-week intervention period and a post intervention assessment. Subsequently, after 4 weeks a follow-up assessment will be conducted. This will allow the evaluation of baseline stability, the evaluation of the short-term durability of treatment effects, and enhanced statistical power by enabling within-subject modeling.

Eligibility criteria will be assessed during intake assessment prior to enrollment. The participants will be randomly allocated to one of the three intervention arms: (1). HIIT; (2). CAT; (3). MA. Stratified block randomization via CASTOR EDC will be used to minimize selection bias. Randomization will be stratified by disease diagnosis (PD or MS), with six blocks of four participants per diagnosis group allocated sequentially over the course of this study.

This study is approved by the medical ethical review board of the Amsterdam UMC (2021.0476). The current study status: enrollment and data collection. Additionally, blood samples will be collected and stored in the HersenFIT study biobank for future biomarker analyses, as approved by the institutional biobank review committee.

This study was prospectively registered at www.ClinicalTrials.gov (ID: NCT05357638).

### 2.2. Participants

A total of 48 participants will be recruited: 24 individuals with PD and 24 with MS. Recruitment will occur through outpatient clinics (Departments of Rehabilitation Medicine, Neurology, and Psychiatry) at Amsterdam UMC, as well as through patient societies and social media platforms.

Inclusion criteria include (1). a clinical diagnosis of PD or progressive MS [54,55,56]; (2). for PD a Hoehn–Yahr stage of <4, indicating mild-to-moderate disability but still physically independent [57]; and for MS an expanded disability status scale (EDSS) of <6, indicating the ability to walk without assistance for at least 200 m) [58]; (3). mild affective disturbances (i.e., hospital anxiety and depression scale (HADS) ≥ 8 for the anxiety and/or depression subscale) [59,60]; (4). able to participate in intensive exercise (i.e., no contra-indications for training according to the American Colleges of Sports Medicine, including no history of heart problems or symptoms including resting electrocardiogram and no other major health issues) [61,62]; (5). sufficient cognitive abilities (Montreal Cognitive Assessment (MoCA) score > 21) [63]; (6). stable medication regimen for at least 4 weeks prior to study enrolment; (7). age ≥ 18 years; (8). able to give informed consent.

Participants are excluded in the case of (1). already participating in a high-intensity exercise program; (2). already participating in an intervention study; (3). an MS relapse 1 month prior to initiation of this study; (4). psychiatric (including suicidal ideation) or musculoskeletal disorders prohibiting participation in intensive exercise.

#### 2.2.1. Safety Monitoring and Withdrawal

Safety procedures will follow the protocol approved by the accredited medical ethical review board. All adverse events are recorded and graded according to the Common Terminology Criteria for Adverse Events. Serious adverse events will be reported within 7 days to the accredited medical ethical review board.

All exercise sessions will be supervised by qualified physiotherapists experienced in managing Parkinson’s disease and multiple sclerosis, ensuring the appropriate handling of disease-specific risks during training.

Participation is voluntary. The participants may withdraw at any time. Those who withdraw before completing the second baseline assessment will be replaced; those who withdraw from the intervention phase will be invited to complete the post intervention and follow-up assessments.

#### 2.2.2. Sample Size

The sample size was calculated based on the primary outcome (HADS total score). Anticipated improvements were 6 points in the HIIT group and 3 points in the CAT group, with an standard deviation (SD) of 6 [64]. Using G*Power (version 3.1) for repeated measures analysis of variance with an alpha of 0.05 and 80% power, a total of 42 participants is required. Accounting for an expected 15% dropout rate, 48 participants will be recruited.

### 2.3. Outcome Assessment

Baseline assessments will include demographic, disease-specific characteristics, and pharmacotherapy (participants will be asked to maintain their existing regimen unless medically required to adjust). The assessor will be blinded to group allocation. Outcome measures will be assessed at baseline, pre intervention (week 4), post intervention (week 12), and follow-up (week 16). An overview is provided in Table 1.

#### 2.3.1. Primary Outcome

The validated HADS will be used to assess symptoms of anxiety and depression [60]. This 14-item self-report tool includes two subscales (HADS-A and HADS-D) each scored from 0 to 21, with higher scores indicating greater severity. A total score (range 0–42) will also be calculated.

#### 2.3.2. Additional Outcomes: Non-Motor Symptoms

In addition to the HADS, affective disturbances will also be monitored using visual analog scales (VAS) [65]. The participants will rate the severity of depression and anxiety using a 100 mm VAS (1 = ‘extremely depressed/anxious’ and 10 = ‘cannot be happier/not anxious’) three times per week.

Cognition will be evaluated with the symbol digit modalities test (processing speed) [66], Stroop color–word test (executive function) [67], and trail making test (task switching) [68]. A VAS will be used to rate concentration.

Fatigue will be assessed with the checklist individual strength (CIS-20r) fatigue subscale. A score ≥35 is indicative of severe fatigue [69]. Sleep quality will be investigated using the insomnia severity index (ISI) [70]. A score of ≥10 is indicative of insomnia. VAS scales (1–10) will assess perceived fatigue and sleep three times per week.

#### 2.3.3. Additional Outcomes: Motor Symptoms

The 10 m walk test (10MWT) and timed up and go (TUG) test will be used to assess functional mobility [71,72]. During the 10MWT participants will be instructed to walk a 10 m marked course at comfortable walking speed and for the TUG test participants will be instructed to rise from the chair, walk 3 m, turn around, return to the chair, and sit down again. The average speed from three trials will be registered together with the average step number.

Dexterity will be assessed using the nine-hole peg test (9HPT) [73]. Participants are instructed to place and remove nine pegs from a pegboard as quickly as possible using the dominant hand and the non-dominant hand separately.

Specifically for participants with PD, prior to motor symptom assessment, time since last dose of levodopa and the participant’s ‘on/off’ medication state will be recorded; this is not applicable for people with MS.

#### 2.3.4. Additional Outcomes: Neuroplasticity and Neurodegeneration

Structural and functional MRI scans will be performed pre and post intervention (i.e., week 4 and week 9) on a Siemens (Erlangen Germany) Vida 3T equipped with a 64-channel head coil. T1-weighted magnetization-prepared rapid gradient echo (MPRAGE), fluid-attenuated inversion recovery (FLAIR), neuromelanin (NM), quantitative susceptibility mapping (QSM), diffusion-weighted imaging (DWI), and resting state functional MRI sequences will be employed to examine brain volumetrics, white matter lesions, the quantification of neuromelanin in the substantia nigra, iron deposits, whiter matter integrity, and resting state functional connectivity, respectively. These measures are incorporated to better understand the potentially underlying neuroprotective mechanisms.

Weekly non-fasting plasma and serum samples (10 mL; aliquot of 0.5 mL) will be collected to measure the concentration of brain-derived neurotrophic factors (BDNFs) [74,75], a marker of neuroplasticity, and neurofilament light (NfL) [76], a marker for axonal damage. Aliquots will be stored at −80 °C until processing for biomarker analysis. BDNF will be measured in serum using the Quanterix Simoa kit; serum NfL with a validated in-house Simoa assay [77]. At first blood draw, additional plasma and serum will be collected for future analyses (10 mL; aliquot of 0.5 mL).

#### 2.3.5. Additional Outcomes

The Nottingham extended activities of daily living scale (NEADL), a self-reported outcome measure to assess instrumental activities of daily living, will be utilized to examine four participation domains: mobility, kitchen, domestic, and leisure activities [78]. The 22-item scale is scored from 0 to 3, with higher scores indicating greater functional independence.

Health-related quality of life will be assessed with the 36-item short-form health survey (SF-36) [79]. Scores are transformed to a 0–100 scale for the different subscales (e.g., physical functioning, mental health, and pain), with higher scores reflecting better perceived quality of life.

The 13-item physical activity scale for individuals with physical disabilities (PASIPD) was used to examine 7-day physical activity in daily life, assessed as the weekly amount of physical activity expressed in metabolic equivalent hours per day (METS) [80]. Changes in PASIPD scores can indicate a shift in habitual activity patterns.

To determine the appropriate training intensities of the HIIT and CAT interventions, a cardiopulmonary exercise test (CPET) will be performed. Peak oxygen uptake (in ml/min and ml/kg/min) and oxygen uptake at the first and second ventilatory threshold, as well as load (in Watt) and heart rate (in bpm), will be registered. The CPET is considered the gold-standard measure of aerobic capacity. To determine the intensity for the MA group, participants in this group will wear a Fitbit Inspire 2 watch. The average daily step count during the baseline period will form the basis of the step count in the intervention period. Daily step count will be monitored during the baseline and intervention period.

### 2.4. Intervention

All interventions will consist of an 8-week individually tailored exercise program based on each participants’ physical fitness level. This duration reflects a balance between effectiveness and feasibility [81]. Detailed descriptions of the interventions are provided in the supplementary Template for Intervention Description and Replication (TIDieR) checklist (Supplementary File S1).

HIIT and CAT will be performed twice weekly for 8 weeks on a cycle ergometer under the supervision of a physiotherapist. The HIIT consists of 5–6 45 s bouts of high-intensity bursts at 85–95% of peak load reached during a CPET, alternating with low-intensity 90 s bouts at an intensity below the first ventilatory threshold. CAT consists of two 20 min bouts at intensities below the first ventilatory threshold interspersed with 2 min breaks.

The participants in the MA group will receive information on physical activity based on the Dutch physical activity guidelines, which recommend engaging in at least 150 min of continuous moderate-to-vigorous-intensity physical activity per week and performing muscle-strengthening exercises on two days per week [82]. To support self-monitoring, each participant will be provided with a Fitbit smartwatch to track daily step counts throughout this study. The average daily step count recorded during the baseline phase will serve as the individualized reference point for their movement goals. During the eight-week intervention period, participants in the MA group will be encouraged to increase their daily step count by approximately 3000 steps on at least five days each week, relative to their personal baseline [83].

### 2.5. Statistical Analysis

Statistical analyses will be conducted using R or Stata, with the significance set at *p* < 0.05 (two-tailed). The primary analysis will follow the intention-to-treat principle and per protocol analysis will be performed as a sensitivity analysis.

Continuous variables will be presented as mean (SD) or median [IQR] values based on normality. Categorical variables will be reported as frequency (%). Normality will be assessed using histograms and Q-Q plots. In case normality assumptions are violated, a log-transformation will be explored.

Baseline characteristics, including age, sex, disease severity, anxiety, depression, and medication use, will be presented for each intervention arm and for each disease type (PD, MS) using descriptive statistics.

Linear mixed models (LMMs) will be used to analyze the longitudinal changes in anxiety and depression across time points (baseline, intervention, and follow-up) and between intervention arms (HIIT, CAT, and MA). These factors will be added to the model as fixed factors. All outcomes will serve as dependent variables in separate models. Participant ID will be added to the model as a random effect to account for intra-individual correlations. Age, sex, medication, and mobility severity (i.e., TUG) will be added to the model as covariates and interaction effects for disease will be investigated. Exploratory subgroup analyses will be conducted if the interaction term is significant. The MA group will be the reference group.

The analyses of secondary outcomes will be exploratory in nature. No formal adjustment for multiple comparisons will be applied; exact *p*-values will be reported, and the results will be interpreted cautiously considering the increased risk of Type I error.

For all primary and secondary outcomes, estimated effects with 95% confidence intervals will be reported from the LMM, alongside standardized effect sizes (Hedges’ g with 95% confidence interval) to facilitate clinical interpretation.

Missing data will be expected, but LMMs are generally robust to missing data. Outliers will be identified using residual diagnostics and sensitivity analyses will be performed with and without outliers to evaluate robustness.

To examine the associations between markers of neuroplasticity and neurodegeneration, and motor and non-motor symptoms, Pearson’s or Spearman’s correlation coefficients will be used depending on normality. Associations will be assessed for changes over time, specifically for the intervention phase.

## 3. Expected Results and Discussion

Exercise therapy is already widely recognized as a valuable rehabilitation tool in PD and MS, which is used to manage motor symptoms, enhance quality of life, and promote social inclusion [24,27]. Recent research also points toward the potential disease-modifying effects of exercise, possibly mediated through neuroplastic and neuroprotective mechanisms [84,85]. The current study is designed to address two main objectives: (1) to identify which of three interventions—HIIT, CAT, or MA—is most effective in improving affective and other non-motor symptoms in PD and MS, (2) to gain insight into the neurobiological mechanisms underlying these effects through biomarker and functional outcome analyses.

Meta-analyses of various exercise modalities (e.g., aerobic and resistance training) show moderate effects on depressive symptoms and small effects on anxiety) compared with control conditions in the general population [86]. These effects appear more pronounced in clinical populations. For individuals with PD, large effects have been reported for depression (SMD = −0.71, 95% CI −0.96 to −0.46) and small-to-moderate effects for anxiety (SMD = 0.39, 95% CI −0.65 to −0.14) [23]. In MS, moderate effects have been found for depression (SMD = 0.37, 95% CI −0.56 to −0.17), while the effects on anxiety were small and not statistically significant (SMD = 0.16, 95% CI −0.50 to 0.19) [87,88]. Based on these previous findings we expect that all three interventions (i.e., HIIT, CAT, and MA) will result in improvements in affective symptoms, with HIIT potentially offering the greatest benefit due to its intensity and time efficiency [89,90].

A strength of this study is its transdiagnostic approach, which focuses on the shared underlying mechanisms and pathways across PD and MS [15,18]. This aligns with an emerging shift in mental health research towards transdiagnostic models, moving beyond rigid diagnostic boundaries to identify common processes and intervention targets [91]. By targeting common pathways such as neuroinflammation or reduced neuroplasticity, this approach has the potential to generate findings that are broadly applicable across neurodegenerative conditions. Additionally, the transdiagnostic framework enables the identification of subgroups with similar symptom profiles across different diagnoses, paving the way for a more personalized and tailored approach to treatment. However, the inclusion of heterogeneous populations, such as individuals with PD and MS, may introduce variability that complicates data interpretation. While focusing on shared mechanisms is a strength, there is a risk of oversimplifying disease-specific features that may be critical for understanding or treating certain symptoms. Another major strength of this study is its mechanistic component. By measuring serum levels of BDNF and NfL, we aim to explore how exercise may modulate neuroplasticity and neurodegeneration. We expect increases in BDNF and potentially reductions in NfL concentrations [45,46,49,50]. Given the pathophysiological differences, responses may diverge between the PD and MS populations. In the evolving landscape of biomarker research, future analyses may also examine other emerging biomarkers, such as but not limited to vascular endothelial growth factor (VEGF), insulin-like Growth Factor 1 (IGF-1), and interleukin-15 (IL-15) [31,92,93], to further investigate the link between immune and neural adaptations to exercise. These biomarker outcomes will be mapped across timepoints and intervention arms, with a detailed figure included to clarify the measurement windows and expected trends.

Beyond the direct comparisons of intervention effectiveness, this study provides an opportunity to examine whether changes in affective symptoms co-occur with motor improvements and neurobiological adaptations. These associative or mediating relationships may offer deeper insight into how exercise exerts multidimensional benefits in neurological populations.

The outcomes of this study could inform the development of tailored exercise-based interventions in clinical practice. Should HIIT prove superior, it may become a preferred modality in future rehabilitation protocols—though further validation in larger cohorts and across diverse patient subgroups would be needed. Future studies should explore for whom, under what conditions, and for how long HIIT remains effective. A long-term follow-up assessment could provide essential data on the sustainability of intervention effects and the practical challenges of maintaining high-intensity exercise. Furthermore, the inclusion of home-based MA acknowledges real-world barriers to structure exercise, offering a flexible and accessible alternative that could enhance adherence and long-term outcomes.

In conclusion, we present the protocol of a transdiagnostic single-blind randomized controlled trail investigating the effects of HIIT on non-motor symptoms, particularly anxiety and depression, compared with CAT and MA in individuals with PD and MS. The secondary objectives include evaluating the impact of exercise on blood and MRI measures of neuroplasticity, neurodegeneration, and overall quality of life. Ultimately, this study addresses a critical gap in the rehabilitation of people with PD or MS by focusing on non-motor symptoms, which have a profound impact on quality of life but are often overshadowed by motor impairments. By adopting a transdiagnostic approach, the findings could inform the development of generalizable, mechanism-driven interventions that transcend disease-specific boundaries.

## Figures and Tables

**Table 1:** Outcome assessment schedule.

	Study Phase	Baseline				Intervention								Follow-Up			
	**Week**	**1**	**2**	**3**	**4**	**5**	**6**	**7**	**8**	**9**	**10**	**11**	**12**	**13**	**14**	**15**	**16**
**Socio-demographic and disease**	Socio-demographics	*															
	Disease characteristics	*															
	Pharmacotherapy	*															
**characteristics**	MDS-UPDRS ^a^ OR EDSS ^b^	*			*									*			*
	PDQ-8 ^a^ OR MSIS ^b^	*			*									*			*
**Primary outcome**	HADS	*			*									*			*
**Non-motor symptoms**	VAS mood	*	*	*	*	*	*	*	*	*	*	*	*	*	*	*	*
	SDMT	*			*									*			*
	SCWT	*			*									*			*
	TMT	*			*									*			*
	VAS ability to concentrate	*	*	*	*	*	*	*	*	*	*	*	*	*	*	*	*
	CIS-20r	*			*									*			*
	VAS fatigue	*	*	*	*	*	*	*	*	*	*	*	*	*	*	*	*
	ISI	*			*									*			*
	VAS sleep	*	*	*	*	*	*	*	*	*	*	*	*	*	*	*	*
**Motor symptoms**	10MWT	*			*									*			*
	TUG	*			*									*			*
	NHPT	*			*									*			*
**Neuroplasticity** **and neurodegeneration**	Plasma and serum ^c^—BDNF and NfL	*	*	*	*	*	*	*	*	*	*	*	*	*			*
	Brain MRI				*									*			
**Additional outcomes**	QoL—SF-36	*			*									*			*
	Participation—NEADI	*			*									*			*
	CPET				*												
	PA-PASIPD	*	*	*	*	*	*	*	*	*	*	*	*	*	*	*	*

* moment of outcome assessment; ^a^ only for people with Parkinson’s disease; ^b^ only for people with multiple sclerosis; ^c^ for participants that perform intervention at home, blood is collected and assessed in weeks 1, 4, 8, 12, and 16. Abbreviations: HADS, hospital anxiety and depression scale; VAS, visual analog scale; MoCA, Montreal Cognitive Assessment; SDMT, symbol digit modalities test; SCWT, Stroop color–word test; TMT, trial making test; CIS-20r, checklist individuals strength 20r; ISI, insomnia severity index; PDQ-8, Parkinson’s disease questionnaire 8-item; MSIS, multiple sclerosis impact scale; SF-36, short form 36-items; MDS-UPDRS, Movement Disorder Society—unified Parkinson’s disease rating scale; EDSS, expanded disability status scale; 10MWT, 10 m walking test; TUG, timed up and go test; NHPT, nine-hole peg test; PASIPD, physical activity scale for individuals with physical disabilities; NEADI, Nottingham extended activities of daily living index; BDNF, brain-derived neurotrophic factor; NfL, neurofilament light; MRI, magnetic resonance imaging; CPET, cardiopulmonary exercise test.

## Data Availability

No new data was created.

## Supplementary Materials

The following supporting information can be downloaded at: https://www.mdpi.com/article/10.3390/brainsci15090945/s1, File S1: TIDieR checklist [94].

## Trial registry

This study was prospectively registered at www.ClinicalTrials.gov (ID: NCT05357638).

## Abbreviations

The following abbreviations are used in this manuscript:

PD	Parkinson’s disease
MS	Multiple sclerosis
CAT	Continuous aerobic training
HIIT	High-intensity interval training
MA	Movement advice
EDSS	Expanded disability status scale
HADS	Hospital anxiety and depression scale
MoCA	Montreal cognitive assessment
HADS-D	Hospital anxiety and depression scale, depression subscale
HADS-A	Hospital anxiety and depression scale, anxiety subscale
HPA axis	Hypothalamic–pituitary–adrenal axis
VAS	Visual analog scale
CIS20r	Checklist individual strength—20r
ISI	Insomnia severity index
10MWT	10 m walk test
TUG	Timed up and go test
9HPT	Nine-hole peg test
MPRAGE	Magnetization-prepared rapid gradient echo
FLAIR	Fluid-attenuated inversion recovery
NM	Neuromelanin
DWI	Diffusion-weighted imaging
BDNF	Brain-derived neurotrophic factor
NfL	Neurofilament light
NEADL	Nottingham extended activities of daily living scale
SF-36	Short-form health survey
PASIPD	Physical activity scale for individuals with physical disabilities
METS	Metabolic equivalents
CPET	Cardiopulmonary exercise test
SD	Standard deviation
IQR	Interquartile range
LMM	Linear mixed model
SMD	Standardized mean difference
95%CI	95% confidence interval
VEGF	Vascular endothelial growth factor
IGF-1	Insulin-like growth factor
IL-15	Interleukin 15

## References

[1] Hirsch E.C., Hunot S. (2009). Neuroinflammation in Parkinson’s disease: A target for neuroprotection?. Lancet Neurol..

[2] Mey G.M., Mahajan K.R., DeSilva T.M. (2023). Neurodegeneration in multiple sclerosis. WIREs Mech. Dis..

[3] Poewe W., Seppi K., Tanner C.M., Halliday G.M., Brundin P., Volkmann J., Schrag A.-E., Lang A.E. (2017). Parkinson disease. Nat. Rev. Dis. Primers.

[4] Bloem B.R., Okun M.S., Klein C. (2021). Parkinson’s disease. Lancet.

[5] Comber L., Galvin R., Coote S. (2017). Gait deficits in people with multiple sclerosis: A systematic review and meta-analysis. Gait Posture.

[6] Mirelman A., Bonato P., Camicioli R., Ellis T.D., Giladi N., Hamilton J.L., Hass C.J., Hausdorff J.M., Pelosin E., Almeida Q.J. (2019). Gait impairments in Parkinson’s disease. Lancet Neurol..

[7] Beiske A.G., Svensson E., Sandanger I., Czujko B., Pedersen E.D., Aarseth J.H., Myhr K.M. (2008). Depression and anxiety amongst multiple sclerosis patients. Eur. J. Neurol..

[8] Braley T.J., Chervin R.D. (2010). Fatigue in multiple sclerosis: Mechanisms, evaluation, and treatment. Sleep.

[9] Langdon D.W. (2011). Cognition in multiple sclerosis. Curr. Opin. Neurol..

[10] Pfeiffer R.F. (2016). Non-motor symptoms in Parkinson’s disease. Park. Relat. Disord..

[11] Boeschoten R.E., Braamse A.M.J., Beekman A.T.F., Cuijpers P., van Oppen P., Dekker J., Uitdehaag B.M.J. (2017). Prevalence of depression and anxiety in Multiple Sclerosis: A systematic review and meta-analysis. J. Neurol. Sci..

[12] Broen M.P., Narayen N.E., Kuijf M.L., Dissanayaka N.N., Leentjens A.F. (2016). Prevalence of anxiety in Parkinson’s disease: A systematic review and meta-analysis. Mov. Disord..

[13] Reijnders J.S., Ehrt U., Weber W.E., Aarsland D., Leentjens A.F. (2008). A systematic review of prevalence studies of depression in Parkinson’s disease. Mov. Disord..

[14] Pontone G.M., Bakker C.C., Chen S., Mari Z., Marsh L., Rabins P.V., Williams J.R., Bassett S.S. (2016). The longitudinal impact of depression on disability in Parkinson disease. Int. J. Geriatr. Psychiatry.

[15] Hussain M., Kumar P., Khan S., Gordon D.K., Khan S. (2020). Similarities Between Depression and Neurodegenerative Diseases: Pathophysiology, Challenges in Diagnosis and Treatment Options. Cureus.

[16] Alvaro P.K., Roberts R.M., Harris J.K. (2013). A Systematic Review Assessing Bidirectionality between Sleep Disturbances, Anxiety, and Depression. Sleep.

[17] Dauwan M., Begemann M.J.H., Slot M.I.E., Lee E.H.M., Scheltens P., Sommer I.E.C. (2021). Physical exercise improves quality of life, depressive symptoms, and cognition across chronic brain disorders: A transdiagnostic systematic review and meta-analysis of randomized controlled trials. J. Neurol..

[18] Husain M. (2017). Transdiagnostic neurology: Neuropsychiatric symptoms in neurodegenerative diseases. Brain.

[19] Dobson R., Giovannoni G. (2019). Multiple sclerosis—A review. Eur. J. Neurol..

[20] Fiest K.M., Walker J.R., Bernstein C.N., Graff L.A., Zarychanski R., Abou-Setta A.M., Patten S.B., Sareen J., Bolton J.M., Marriott J.J. (2016). Systematic review and meta-analysis of interventions for depression and anxiety in persons with multiple sclerosis. Mult. Scler. Relat. Disord..

[21] Pontone G.M., Mills K.A. (2021). Optimal Treatment of Depression and Anxiety in Parkinson’s Disease. Am. J. Geriatr. Psychiatry.

[22] Ryan M., Eatmon C.V., Slevin J.T. (2019). Drug treatment strategies for depression in Parkinson disease. Expert Opin. Pharmacother..

[23] Costa V., Prati J.M., de Oliveira Barreto Suassuna A., Souza Silva Brito T., Frigo da Rocha T., Gianlorenco A.C. (2024). Physical Exercise for Treating the Anxiety and Depression Symptoms of Parkinson’s Disease: Systematic Review and Meta-Analysis. J. Geriatr. Psychiatry Neurol..

[24] Motl R.W., Sandroff B.M., Kwakkel G., Dalgas U., Feinstein A., Heesen C., Feys P., Thompson A.J. (2017). Exercise in patients with multiple sclerosis. Lancet Neurol..

[25] Silic P., Motl R.W., Duffecy J. (2023). Multiple sclerosis and anxiety: Is there an untapped opportunity for exercise?. Mult. Scler. Relat. Disord..

[26] Strohle A. (2009). Physical activity, exercise, depression and anxiety disorders. J. Neural. Transm..

[27] Mak M.K., Wong-Yu I.S., Shen X., Chung C.L. (2017). Long-term effects of exercise and physical therapy in people with Parkinson disease. Nat. Rev. Neurol..

[28] Johansson M.E., Cameron I.G.M., Van der Kolk N.M., de Vries N.M., Klimars E., Toni I., Bloem B.R., Helmich R.C. (2022). Aerobic Exercise Alters Brain Function and Structure in Parkinson’s Disease: A Randomized Controlled Trial. Ann. Neurol..

[29] Stellmann J.P., Maarouf A., Schulz K.H., Baquet L., Pottgen J., Patra S., Penner I.K., Gellissen S., Ketels G., Besson P. (2020). Aerobic Exercise Induces Functional and Structural Reorganization of CNS Networks in Multiple Sclerosis: A Randomized Controlled Trial. Front. Hum. Neurosci..

[30] Karlsen T., Aamot I.L., Haykowsky M., Rognmo O. (2017). High Intensity Interval Training for Maximizing Health Outcomes. Prog. Cardiovasc. Dis..

[31] Atakan M.M., Li Y., Kosar S.N., Turnagol H.H., Yan X. (2021). Evidence-Based Effects of High-Intensity Interval Training on Exercise Capacity and Health: A Review with Historical Perspective. Int. J. Environ. Res. Public Health.

[32] Foster C., Casado A., Bok D., Hofmann P., Bakken M., Tjelta A., Manso J., Boullosa D., de Koning J. (2025). History and perspectives on interval training in sport, health, and disease. Appl. Physiol. Nutr. Metab..

[33] Gallo P.M. (2021). High-intensity interval training for neurodegenerative conditions: Indications and recommendations for exercise programming. ACSM’s Health Fit. J..

[34] Campbell E., Coulter E.H., Paul L. (2018). High intensity interval training for people with multiple sclerosis: A systematic review. Mult. Scler. Relat. Disord..

[35] Harpham C., Gunn H., Marsden J., Connolly L. (2023). The feasibility, safety, physiological and clinical effects of high-intensity interval training for people with Parkinson’s: A systematic review and meta-analysis. Aging Clin. Exp. Res..

[36] Youssef H., Gonul M.N., Sobeeh M.G., Akar K., Feys P., Cuypers K., Vural A. (2024). Is High-Intensity Interval Training More Effective Than Moderate Continuous Training in Rehabilitation of Multiple Sclerosis: A Comprehensive Systematic Review and Meta-analysis. Arch. Phys. Med. Rehabil..

[37] Martland R., Mondelli V., Gaughran F., Stubbs B. (2020). Can high-intensity interval training improve physical and mental health outcomes? A meta-review of 33 systematic reviews across the lifespan. J. Sports Sci..

[38] Plag J., Schmidt-Hellinger P., Klippstein T., Mumm J.L.M., Wolfarth B., Petzold M.B., Strohle A. (2020). Working out the worries: A randomized controlled trial of high intensity interval training in generalized anxiety disorder. J. Anxiety Disord..

[39] Gaia J.W.P., Schuch F.B., Ferreira R.W., Souza E.L., Ferreira V.M.S., Pires D.A. (2024). Effects of high-intensity interval training on depressive and anxiety symptoms in healthy individuals: A systematic review and meta-analysis of randomized clinical trials. Scand. J. Med. Sci. Sports.

[40] Zare N., Bishop D.J., Levinger I., Febbraio M.A., Broatch J.R. (2025). Exercise intensity matters: A review on evaluating the effects of aerobic exercise intensity on muscle-derived neuroprotective myokines. Alzheimers Dement..

[41] Zhang X., Zong B., Zhao W., Li L. (2021). Effects of Mind-Body Exercise on Brain Structure and Function: A Systematic Review on MRI Studies. Brain Sci..

[42] Zheng G., Ye B., Zheng Y., Xiong Z., Xia R., Qiu P., Tao J., Chen L. (2019). The effects of exercise on the structure of cognitive related brain regions: A meta-analysis of functional neuroimaging data. Int. J. Neurosci..

[43] Zhang W., Zhou C., Chen A. (2024). A systematic review and meta-analysis of the effects of physical exercise on white matter integrity and cognitive function in older adults. Geroscience.

[44] Bray N.W., Pieruccini-Faria F., Bartha R., Doherty T.J., Nagamatsu L.S., Montero-Odasso M. (2021). The effect of physical exercise on functional brain network connectivity in older adults with and without cognitive impairment. A systematic review. Mech. Ageing Dev..

[45] Walsh J.J., Tschakovsky M.E. (2018). Exercise and circulating BDNF: Mechanisms of release and implications for the design of exercise interventions. Appl. Physiol. Nutr. Metab..

[46] Gomez-Pinilla F., Ying Z., Roy R.R., Molteni R., Edgerton V.R. (2002). Voluntary exercise induces a BDNF-mediated mechanism that promotes neuroplasticity. J. Neurophysiol..

[47] Martinowich K., Manji H., Lu B. (2007). New insights into BDNF function in depression and anxiety. Nat. Neurosci..

[48] Ruiz-Gonzalez D., Hernandez-Martinez A., Valenzuela P.L., Morales J.S., Soriano-Maldonado A. (2021). Effects of physical exercise on plasma brain-derived neurotrophic factor in neurodegenerative disorders: A systematic review and meta-analysis of randomized controlled trials. Neurosci. Biobehav. Rev..

[49] Kaagman D.G.M., van Wegen E.E.H., Cignetti N., Rothermel E., Vanbellingen T., Hirsch M.A. (2024). Effects and Mechanisms of Exercise on Brain-Derived Neurotrophic Factor (BDNF) Levels and Clinical Outcomes in People with Parkinson’s Disease: A Systematic Review and Meta-Analysis. Brain Sci..

[50] Shobeiri P., Karimi A., Momtazmanesh S., Teixeira A.L., Teunissen C.E., van Wegen E.E.H., Hirsch M.A., Yekaninejad M.S., Rezaei N. (2022). Exercise-induced increase in blood-based brain-derived neurotrophic factor (BDNF) in people with multiple sclerosis: A systematic review and meta-analysis of exercise intervention trials. PLoS ONE.

[51] Gomes E.S.A., Van den Heuvel O.A., Rietberg M.B., De Groot V., Hirsch M.A., Van de Berg W.D.J., Jaspers R.T., Vriend C., Vanbellingen T., Van Wegen E.E.H. (2023). (HIIT-The Track) High-Intensity Interval Training for People with Parkinson’s Disease: Individual Response Patterns of (Non-)Motor Symptoms and Blood-Based Biomarkers-A Crossover Single-Case Experimental Design. Brain Sci..

[52] Kim R., Choi S., Kang N., Park K., Shin H., Lee H., Lee H., Jun J.S., Jeon B., Byun K. (2024). Effects of high-intensity interval training and moderate-intensity continuous training on non-motor symptoms in patients with Parkinson’s disease: A randomised pilot trial. J. Neurol. Neurosurg. Psychiatry.

[53] Ellis T., Boudreau J.K., DeAngelis T.R., Brown L.E., Cavanaugh J.T., Earhart G.M., Ford M.P., Foreman K.B., Dibble L.E. (2013). Barriers to exercise in people with Parkinson disease. Phys. Ther..

[54] Thompson A.J., Banwell B.L., Barkhof F., Carroll W.M., Coetzee T., Comi G., Correale J., Fazekas F., Filippi M., Freedman M.S. (2018). Diagnosis of multiple sclerosis: 2017 revisions of the McDonald criteria. Lancet Neurol..

[55] Tolosa E., Garrido A., Scholz S.W., Poewe W. (2021). Challenges in the diagnosis of Parkinson’s disease. Lancet Neurol..

[56] Lublin F.D., Reingold S.C., Cohen J.A., Cutter G.R., Sorensen P.S., Thompson A.J., Wolinsky J.S., Balcer L.J., Banwell B., Barkhof F. (2014). Defining the clinical course of multiple sclerosis: The 2013 revisions. Neurology.

[57] Goetz C.G., Poewe W., Rascol O., Sampaio C., Stebbins G.T., Counsell C., Giladi N., Holloway R.G., Moore C.G., Wenning G.K. (2004). Movement Disorder Society Task Force report on the Hoehn and Yahr staging scale: Status and recommendations. Mov. Disord..

[58] Kurtzke J.F. (1983). Rating neurologic impairment in multiple sclerosis: An expanded disability status scale (EDSS). Neurology.

[59] Bjelland I., Dahl A.A., Haug T.T., Neckelmann D. (2002). The validity of the Hospital Anxiety and Depression Scale. An updated literature review. J. Psychosom. Res..

[60] Zigmond A.S., Snaith R.P. (1983). The hospital anxiety and depression scale. Acta Psychiatr. Scand..

[61] Thompson P.D., Arena R., Riebe D., Pescatello L.S. (2013). ACSM’s new preparticipation health screening recommendations from ACSM’s guidelines for exercise testing and prescription. Curr. Sports Med. Rep..

[62] Bille K., Figueiras D., Schamasch P., Kappenberger L., Brenner J.I., Meijboom F.J., Meijboom E.J. (2006). Sudden cardiac death in athletes: The Lausanne Recommendations. Eur. J. Prev. Cardiol..

[63] Nasreddine Z.S., Phillips N.A., Bédirian V., Charbonneau S., Whitehead V., Collin I., Cummings J.L., Chertkow H. (2005). The Montreal Cognitive Assessment, MoCA: A brief screening tool for mild cognitive impairment. J. Am. Geriatr. Soc..

[64] Nieuwboer A., Kwakkel G., Rochester L., Jones D., van Wegen E., Willems A.M., Chavret F., Hetherington V., Baker K., Lim I. (2007). Cueing training in the home improves gait-related mobility in Parkinson’s disease: The RESCUE trial. J. Neurol. Neurosurg. Psychiatry.

[65] Wewers M.E., Lowe N.K. (1990). A critical review of visual analogue scales in the measurement of clinical phenomena. Res. Nurs. Health.

[66] Smith A. (1973). Symbol digit modalities test. The Clinical Neuropsychologist.

[67] Golden C., Freshwater S.M., Golden Z. (1978). Stroop Color and Word Test.

[68] Bowie C.R., Harvey P.D. (2006). Administration and interpretation of the Trail Making Test. Nat. Protoc..

[69] Worm-Smeitink M., Gielissen M., Bloot L., van Laarhoven H.W.M., van Engelen B.G.M., van Riel P., Bleijenberg G., Nikolaus S., Knoop H. (2017). The assessment of fatigue: Psychometric qualities and norms for the Checklist individual strength. J. Psychosom. Res..

[70] Morin C.M., Belleville G., Bélanger L., Ivers H. (2011). The Insomnia Severity Index: Psychometric indicators to detect insomnia cases and evaluate treatment response. Sleep.

[71] Peters D.M., Fritz S.L., Krotish D.E. (2013). Assessing the reliability and validity of a shorter walk test compared with the 10-Meter Walk Test for measurements of gait speed in healthy, older adults. J. Geriatr. Phys. Ther..

[72] Herman T., Giladi N., Hausdorff J.M. (2011). Properties of the ‘timed up and go’test: More than meets the eye. Gerontology.

[73] Feys P., Lamers I., Francis G., Benedict R., Phillips G., LaRocca N., Hudson L.D., Rudick R., Consortium M.S.O.A. (2017). The Nine-Hole Peg Test as a manual dexterity performance measure for multiple sclerosis. Mult. Scler. J..

[74] Autry A.E., Monteggia L.M. (2012). Brain-derived neurotrophic factor and neuropsychiatric disorders. Pharmacol. Rev..

[75] Zuccato C., Cattaneo E. (2009). Brain-derived neurotrophic factor in neurodegenerative diseases. Nat. Rev. Neurol..

[76] Gaetani L., Blennow K., Calabresi P., Di Filippo M., Parnetti L., Zetterberg H. (2019). Neurofilament light chain as a biomarker in neurological disorders. J. Neurol. Neurosurg. Psychiatry.

[77] Kuhle J., Barro C., Disanto G., Mathias A., Soneson C., Bonnier G., Yaldizli O., Regeniter A., Derfuss T., Canales M. (2016). Serum neurofilament light chain in early relapsing remitting MS is increased and correlates with CSF levels and with MRI measures of disease severity. Mult. Scler..

[78] das Nair R.B., Moreton B.J., Lincoln N.B. (2011). Rasch analysis of the Nottingham extended activities of daily living scale. J. Rehabil. Med..

[79] Ware J.E., Sherbourne C.D. (1992). The MOS 36-item short-form health survey (SF-36). I. Conceptual framework and item selection. Med. Care.

[80] Washburn R.A., Zhu W., McAuley E., Frogley M., Figoni S.F. (2002). The physical activity scale for individuals with physical disabilities: Development and evaluation. Arch. Phys. Med. Rehabil..

[81] Huang G., Wang R., Chen P., Huang S.C., Donnelly J.E., Mehlferber J.P. (2016). Dose-response relationship of cardiorespiratory fitness adaptation to controlled endurance training in sedentary older adults. Eur. J. Prev. Cardiol..

[82] Gezondheidsraad Beweegrichtlijnen 2017—Samenvatting. https://www.gezondheidsraad.nl/documenten/adviezen/2017/08/22/beweegrichtlijnen-2017.

[83] Paluch A.E., Bajpai S., Bassett D.R., Carnethon M.R., Ekelund U., Evenson K.R., Galuska D.A., Jefferis B.J., Kraus W.E., Lee I.M. (2022). Daily steps and all-cause mortality: A meta-analysis of 15 international cohorts. Lancet Public Health.

[84] Diechmann M.D., Campbell E., Coulter E., Paul L., Dalgas U., Hvid L.G. (2021). Effects of Exercise Training on Neurotrophic Factors and Subsequent Neuroprotection in Persons with Multiple Sclerosis-A Systematic Review and Meta-Analysis. Brain Sci..

[85] Hirsch M.A., Iyer S.S., Sanjak M. (2016). Exercise-induced neuroplasticity in human Parkinson’s disease: What is the evidence telling us?. Parkinsonism Relat. Disord..

[86] Wegner M., Helmich I., Machado S., Nardi A.E., Arias-Carrion O., Budde H. (2014). Effects of exercise on anxiety and depression disorders: Review of meta- analyses and neurobiological mechanisms. CNS Neurol. Disord. Drug Targets.

[87] Dalgas U., Stenager E., Sloth M., Stenager E. (2015). The effect of exercise on depressive symptoms in multiple sclerosis based on a meta-analysis and critical review of the literature. Eur. J. Neurol..

[88] Gascoyne C., Karahalios A., Demaneuf T., Marck C. (2020). Effect of Exercise Interventions on Anxiety in People with Multiple Sclerosis: A Systematic Review and Meta-analysis. Int. J. MS Care.

[89] Borrega-Mouquinho Y., Sanchez-Gomez J., Fuentes-Garcia J.P., Collado-Mateo D., Villafaina S. (2021). Effects of High-Intensity Interval Training and Moderate-Intensity Training on Stress, Depression, Anxiety, and Resilience in Healthy Adults During Coronavirus Disease 2019 Confinement: A Randomized Controlled Trial. Front. Psychol..

[90] Gu T., Hao P., Chen P., Wu Y. (2022). A Systematic Review and Meta-Analysis of the Effectiveness of High-Intensity Interval Training in People with Cardiovascular Disease at Improving Depression and Anxiety. Evid. Based Complement. Alternat. Med..

[91] Dalgleish T., Black M., Johnston D., Bevan A. (2020). Transdiagnostic approaches to mental health problems: Current status and future directions. J. Consult. Clin. Psychol..

[92] Munoz A., Correa C.L., Lopez-Lopez A., Costa-Besada M.A., Diaz-Ruiz C., Labandeira-Garcia J.L. (2018). Physical Exercise Improves Aging-Related Changes in Angiotensin, IGF-1, SIRT1, SIRT3, and VEGF in the Substantia Nigra. J. Gerontol. A Biol. Sci. Med. Sci..

[93] Najafi P., Hadizadeh M., Cheong J.P.G., Mohafez H., Abdullah S. (2022). Cytokine Profile in Patients with Multiple Sclerosis Following Exercise: A Systematic Review of Randomized Clinical Trials. Int. J. Environ. Res. Public Health.

[94] Williams N. (2017). The Borg rating of perceived exertion (RPE) scale. Occup. Med..

