# Supplementary material for: HIITing Anxiety and Depression in Parkinson’s Disease and Multiple Sclerosis—A Study Protocol of a Transdiagnostic Randomized Controlled Trial (HersenFIT)
Source: Brain Sci. 2025 Aug 29;15(9):945. doi: 10.3390/brainsci15090945 (PMC12468372; doi:10.3390/brainsci15090945)
Supplement: Supplementary file 1 [file brainsci-15-00945-s001.zip › brainsci-3791585-supplementary.pdf]

Supplement - TIDieR checklist - HersenFIT

Template for intervention description and replication

Content:

1. **High-intensity interval training**
2. **Continuous aerobic training**
3. **Movement advice**

1. High-intensity interval training

1.1 Why

High-Intensity Interval Training (HIIT) is one of three interventions evaluated in the HersenFIT study, which investigates the effects of exercise therapy on motor and non-motor symptoms, as well as neuroplasticity, in individuals with Parkinson's disease (PD) and progressive multiple sclerosis (MS). The primary outcome of the study is the level of anxiety and depression, measured using the Hospital Anxiety and Depression Scale (HADS) [1].

Exercise therapy has demonstrated beneficial effects on motor function in both PD and MS [2,3]. Emerging evidence also suggests that it may improve non-motor symptoms—such as anxiety and depression—and positively influence neuroplasticity [4-8]. However, these effects, particularly on non-motor symptoms and brain plasticity, remain insufficiently understood.

Various exercise modalities have been studied, with resistance and endurance training among the most implemented. Notably, endurance-based interventions have shown potential in alleviating anxiety and depression. Traditionally, endurance training is delivered through continuous low-to-moderate intensity sessions. Recently, however, more engaging and time-efficient alternatives such as HIIT have gained attention [9-11]. Despite this interest, comparative studies between different exercise modalities remain limited.

The HersenFIT study addresses this gap by comparing the effects of HIIT, continuous aerobic training, and movement advice on anxiety and depression (primary outcomes), as well as a broader range of non-motor and motor symptoms and measures of neuroplasticity.

1.2 What

HIIT training focusses on high-intensity burst of exercise interspersed by low-intensity rest. The HIIT intervention is performed on a cycle ergometer. Every training will start with 5-minutes warm-up at a low intensity. Then the first bout of three sets of intervals will start, followed by a relative rest of 5-minutes at low intensity. Subsequently a second set of two to three intervals will be performed, followed by a 5-minute cool down at low intensity.

The exact duration and intensity in both Watt and heart rate are provided for each participant in a training-log. See example in table 1.

Table 1. Example of one high-intensity interval training in training log participants

Training	Stage	Duration	Total duration	Intensity Watt	Intensity Heart rate	Borg rating of perceived exertion*
Date:	Warm-up	5 min	5 min	70	100	9
	Rest	90 sec	6,5 min	85	135	10
	Interval	45 sec	7 min 15'	200	180	14
	Rest	90 sec	8 min 45'	85	135	10
	Interval	45 sec	9 min 30'	200	180	14
	Rest	90 sec	11 min	85	135	10
	Interval	45 sec	11 min 45'	200	180	15
	Relative rest	5 min	16 min 45'	70	100	9
	Rest	90 sec	18 min 15'	85	135	10
	Interval	45 sec	19 min	200	180	14
	Rest	90 sec	20 min 30'	85	135	10
	Interval	45 sec	21 min 15'	200	180	15
	Rest	90 sec	22 min 45'	85	135	10
	Cool down	5 min	27 min 45'	70	100	9

* Patient-reported outcome measure.

1.3 Who provided

The HIIT program is supervised by a physiotherapist. The physiotherapist is trained in the specific HIIT program by one of the research team members, according to the study protocol. In case of questions, physiotherapists can easily contact the research team by telephone or email.

1.4 How

The HIIT training can be performed in small physiotherapist-guided groups of maximally 8 participants or alone with one-to-one guidance of a physiotherapist. The instructions will be given face-to-face, and participants also have their own training log with basic instructions.

1.5 Where

The intervention can be performed in the fitness center of the department of Rehabilitation Medicine, at the Amsterdam UMC, location VUmc or at a local physiotherapy practice.

1.6 When and how much

The intervention is performed twice a week for 8 weeks of approximately 30 minutes. The training intensity, volumes and rest are provided in table 2.

Table 2. Training volume and intensity

Week	Interval intensity (% of peak Watt)	Volume	Interval duration	Rest duration
1	85%	5 intervals	45 sec	90 sec
2	88%	5 intervals	45 sec	90 sec
3	91%	5 intervals	45 sec	90 sec
4	94%	5 intervals	45 sec	90 sec
5	85%	6 intervals	45 sec	90 sec
6	88%	6 intervals	45 sec	90 sec
7	91%	6 intervals	45 sec	90 sec
8	94%	6 intervals	45 sec	90 sec

1.7 Tailoring and modifications

The intensity of the HIIT program is based on the peak load in Watt (peak Watt) measured during a cardiopulmonary exercise test. During the intervention the Borg rating of perceived exertion is assessed during for all sessions [12]. Based on the physiotherapist experiences together with the Borg rating of perceived exertion the interval intensity can be either increased or decreased, but interval durations remain stable. All modifications to the original program will be documented in the training log, including reason for modification.

1.10 How well

During every session participant will record their training sessions in a training log, for example see table 1. Participants will register their Borg rating of perceived exertion for every phase of the session, and modifications in interval intensity will be reported as well.

2. Continuous aerobic training

2.1 Why

Continuous aerobic training (CAT) is one of three interventions evaluated in the HersenFIT study, which investigates the effects of exercise therapy on motor and non-motor symptoms, as well as neuroplasticity, in individuals with Parkinson's disease (PD) and progressive multiple sclerosis (MS). The primary outcome of the study is the level of anxiety and depression, measured using the Hospital Anxiety and Depression Scale (HADS) [1].

Exercise therapy has demonstrated beneficial effects on motor function in both PD and MS [2,3]. Emerging evidence also suggests that it may improve non-motor symptoms—such as anxiety and depression—and positively influence neuroplasticity [4-8]. However, these effects, particularly on non-motor symptoms and brain plasticity, remain insufficiently understood.

Various exercise modalities have been studied, with resistance and endurance training among the most implemented. Notably, endurance-based interventions have shown potential in alleviating anxiety and depression. Traditionally, endurance training is delivered through continuous low-to-moderate intensity sessions. Recently, however, more engaging and time-efficient alternatives such as high-intensity interval training (HIIT) have gained attention [9-11]. Despite this interest, comparative studies between different exercise modalities remain limited.

The HersenFIT study addresses this gap by comparing the effects of HIIT, CAT, and movement advice on anxiety and depression (primary outcomes), as well as a broader range of non-motor and motor symptoms and measures of neuroplasticity.

2.2 What

CAT focusses on a longer bout of low-to-moderate intensity exercise. The CAT intervention is performed on a cycle ergometer. Every training will start with a 5-minute warm-up phase at a low intensity, followed by two 20-minute low-to-moderate intensity continuous exercise bouts interspersed by a relative rest period of 5 minutes and completed with a 5-minute cool down stage. For an example see Table 3.

Table 3. Example of one continuous aerobic training in the participant training log

Training	Stage	Duration	Total duration	Intensity Watt	Intensity Heart rate	Borg rating of perceived exertion*
Date:	Warm-up	5 min	5 min	15	100	9
	CAT	20 min	25 min	30	135	12
	Rest	2 min	27 min	15	100	10
	CAT	20 min	47 min	30	135	12
	Cool down	5 min	52 min'	15	100	10

* Patient-reported outcome measure.

2.3 Who provided

The CAT program is supervised by a physiotherapist. The physiotherapist is trained in the specific CAT program by one of the research team members, according to the study protocol. In case of questions, physiotherapists can easily contact the research team by telephone or email.

2.5 How

The CAT training can be performed in small physiotherapist-guided groups of maximally 8 participants or alone with one-to-one guidance of a physiotherapist. The instructions will be given face-to-face, and participants also have their own training log with basic instructions.

2.6 Where
The intervention can be performed in the fitness center of the department of Rehabilitation Medicine, at the Amsterdam UMC, location VUmc or at a local physiotherapy practice.

2.7 When and how much
The intervention is performed twice a week for 8 weeks for approximately 50 minutes. The intensity is just below the first ventilatory threshold. Every training will consist of the same stages as shown in Table 3: 5-minute warm-up 20 minutes CAT, 2 minutes relative rest, 20 minutes CAT and 5-minute cool down.

2.8 Tailoring and modifications
The intensity of the CAT program is based on the load in Watt (peak Watt) at the first ventilatory threshold measured during a cardiopulmonary exercise test. During the intervention the Borg rating of perceived exertion is assessed during for all sessions [12]. Based on the physiotherapist experiences together with the Borg rating of perceived exertion the interval intensity can be either increased or decreased, but CAT durations remain stable. All modifications to the original program will be documented in the training log, including reason for modification.

2.10 How well
During every session participant will record their training sessions in a training log, for example see table 3. Participants will register their Borg rating of perceived exertion for every phase of the session, and modifications in interval intensity will be reported as well.

3. Movement advice

3.1 Why

Movement advice (MA) is one of three interventions evaluated in the HersenFIT study, which investigates the effects of exercise therapy on motor and non-motor symptoms, as well as neuroplasticity, in individuals with Parkinson's disease (PD) and progressive multiple sclerosis (MS). The primary outcome of the study is the level of anxiety and depression, measured using the Hospital Anxiety and Depression Scale (HADS) [1].

Exercise therapy has demonstrated beneficial effects on motor function in both PD and MS [2,3]. Emerging evidence also suggests that it may improve non-motor symptoms—such as anxiety and depression—and positively influence neuroplasticity [4-8]. However, these effects, particularly on non-motor symptoms and brain plasticity, remain insufficiently understood.

Various exercise modalities have been studied, with resistance and endurance training among the most implemented. Notably, endurance-based interventions have shown potential in alleviating anxiety and depression. Traditionally, endurance training is delivered through continuous low-to-moderate intensity sessions. Recently, however, more engaging and time-efficient alternatives such as high intensity interval training (HIIT) have gained attention [9-11]. Despite this interest, comparative studies between different exercise modalities remain limited.

The HersenFIT study addresses this gap by comparing the effects of HIIT, continuous aerobic training, and MA on anxiety and depression (primary outcomes), as well as a broader range of non-motor and motor symptoms and measures of neuroplasticity.

3.2 What

Participants in the MA group are provided with information about the Dutch guidelines for physical activity, the information will be provided on a flyer (see example in Figure 1). Furthermore, the group will wear a Fitbit watch to monitor daily step count. Participants are advised to increase their step count by 3000 steps on at least 5 days in the week during the intervention period. A increase of 3000 septs is approximated to result in 30 minutes of light-to-moderate physical activity an 5 days which sums up to approximately 150 minutes of physical activity.

3.3 Who provided and how performed

The movement advice is given by one of the members of the research team, either a human movement scientist and/or physiotherapist. The intervention itself will be performed by the participants themselves without supervision from home.

3.4 Where

The intervention will be performed in the participants own home environment.

3.5 When and how much

Movement advice will be performed for 8-weeks. During the 4-week extended baseline period an average daily step count will be established. A personal step count was then determined as 3000 extra steps in addition to the average daily step count. During the intervention period, participants were required to reach this step count on at least 5 days a week. The steps can be performed during the whole day, but during the movement advice session participants are stimulated to do this in bouts of at least 10 minutes.

3.8 Tailoring and modifications

The step count is based on baseline daily average step count. During the intervention period participants can always contact the research team in case of any questions. Furthermore, the research team was able to see the

daily step count during the intervention period for every day. In case recordings showed that participants did not reach their step count, the research team contacted the participants to encourage them.

3.10 How well

Participants wore a Fitbit smartwatch that registered step count every day. The daily step count was recorded as well as the number of days a week once participants reached their personal goal. Participants that reached their daily step count on at least 5 days were fully adherent to the program.

References

1. Zigmond, A.S.; Snaith, R.P. The hospital anxiety and depression scale. *Acta Psychiatr Scand* **1983**, *67*, 361-370, doi:10.1111/j.1600-0447.1983.tb09716.x.
2. Mak, M.K.; Wong-Yu, I.S.; Shen, X.; Chung, C.L. Long-term effects of exercise and physical therapy in people with Parkinson disease. *Nat Rev Neurol* **2017**, *13*, 689-703, doi:10.1038/nrneurol.2017.128.
3. Motl, R.W.; Sandroff, B.M.; Kwakkel, G.; Dalgas, U.; Feinstein, A.; Heesen, C.; Feys, P.; Thompson, A.J. Exercise in patients with multiple sclerosis. *Lancet Neurol* **2017**, *16*, 848-856, doi:10.1016/S1474-4422(17)30281-8.
4. Costa, V.; Prati, J.M.; de Oliveira Barreto Suassuna, A.; Souza Silva Brito, T.; Frigo da Rocha, T.; Gianlorenco, A.C. Physical Exercise for Treating the Anxiety and Depression Symptoms of Parkinson's Disease: Systematic Review and Meta-Analysis. *J Geriatr Psychiatry Neurol* **2024**, *37*, 415-435, doi:10.1177/08919887241237223.
5. Dalgas, U.; Stenager, E.; Sloth, M.; Stenager, E. The effect of exercise on depressive symptoms in multiple sclerosis based on a meta-analysis and critical review of the literature. *Eur J Neurol* **2015**, *22*, 443-e434, doi:10.1111/ene.12576.
6. Fiest, K.M.; Walker, J.R.; Bernstein, C.N.; Graff, L.A.; Zarychanski, R.; Abou-Setta, A.M.; Patten, S.B.; Sareen, J.; Bolton, J.M.; Marriott, J.J.; et al. Systematic review and meta-analysis of interventions for depression and anxiety in persons with multiple sclerosis. *Mult Scler Relat Disord* **2016**, *5*, 12-26, doi:10.1016/j.msard.2015.10.004.
7. Gascoyne, C.; Karahalios, A.; Demaneuf, T.; Marck, C. Effect of Exercise Interventions on Anxiety in People with Multiple Sclerosis: A Systematic Review and Meta-analysis. *Int J MS Care* **2020**, *22*, 103-109, doi:10.7224/1537-2073.2019-009R.
8. Hirsch, M.A.; Iyer, S.S.; Sanjak, M. Exercise-induced neuroplasticity in human Parkinson's disease: What is the evidence telling us? *Parkinsonism Relat Disord* **2016**, *22 Suppl 1*, S78-81, doi:10.1016/j.parkreldis.2015.09.030.
9. Atakan, M.M.; Li, Y.; Kosar, S.N.; Turnagol, H.H.; Yan, X. Evidence-Based Effects of High-Intensity Interval Training on Exercise Capacity and Health: A Review with Historical Perspective. *Int J Environ Res Public Health* **2021**, *18*, doi:10.3390/ijerph18137201.
10. Foster, C.; Casado, A.; Bok, D.; Hofmann, P.; Bakken, M.; Tjelta, A.; Manso, J.; Boullosa, D.; de Koning, J. History and perspectives on interval training in sport, health, and disease. *Appl Physiol Nutr Metab* **2025**, *50*, 1-16, doi:10.1139/apnm-2023-0611.
11. Karlsen, T.; Aamot, I.L.; Haykowsky, M.; Rognmo, O. High Intensity Interval Training for Maximizing Health Outcomes. *Prog Cardiovasc Dis* **2017**, *60*, 67-77, doi:10.1016/j.pcad.2017.03.006.
12. Williams, N. The Borg rating of perceived exertion (RPE) scale. *Occupational medicine* **2017**, *67*, 404-405.
